# GLP-1 mediated diuresis and natriuresis are blunted in heart failure and restored by selective afferent renal denervation

**DOI:** 10.1186/s12933-020-01029-0

**Published:** 2020-05-08

**Authors:** Kenichi Katsurada, Shyam S. Nandi, Hong Zheng, Xuefei Liu, Neeru M. Sharma, Kaushik P. Patel

**Affiliations:** 1 Department of Cellular and Integrative Physiology, University of Nebraska Medical Center, 985850 Nebraska Medical Center, Omaha, NE 68198-5850 USA; 2 Division of Basic Biomedical Sciences, Sanford School of Medicine of the University of South Dakota, Vermillion, SD USA

**Keywords:** Glucagon-like peptide-1, Renal afferent, Sympathetic nerve activity, Sodium and water homeostasis, Heart failure

## Abstract

**Background:**

Glucagon-like peptide-1 (GLP-1) induces diuresis and natriuresis. Previously we have shown that GLP-1 activates afferent renal nerve to increase efferent renal sympathetic nerve activity that negates the diuresis and natriuresis as a negative feedback mechanism in normal rats. However, renal effects of GLP-1 in heart failure (HF) has not been elucidated. The present study was designed to assess GLP-1-induced diuresis and natriuresis in rats with HF and its interactions with renal nerve activity.

**Methods:**

HF was induced in rats by coronary artery ligation. The direct recording of afferent renal nerve activity (ARNA) with intrapelvic injection of GLP-1 and total renal sympathetic nerve activity (RSNA) with intravenous infusion of GLP-1 were performed. GLP-1 receptor expression in renal pelvis, densely innervated by afferent renal nerve, was assessed by real-time PCR and western blot analysis. In separate group of rats after coronary artery ligation selective afferent renal denervation (A-RDN) was performed by periaxonal application of capsaicin, then intravenous infusion of GLP-1-induced diuresis and natriuresis were evaluated.

**Results:**

In HF, compared to sham-operated control; (1) response of increase in ARNA to intrapelvic injection of GLP-1 was enhanced (3.7 ± 0.4 vs. 2.0 ± 0.4 µV s), (2) GLP-1 receptor expression was increased in renal pelvis, (3) response of increase in RSNA to intravenous infusion of GLP-1 was enhanced (132 ± 30% vs. 70 ± 16% of the baseline level), and (4) diuretic and natriuretic responses to intravenous infusion of GLP-1 were blunted (urine flow 53.4 ± 4.3 vs. 78.6 ± 4.4 µl/min/gkw, sodium excretion 7.4 ± 0.8 vs. 10.9 ± 1.0 µEq/min/gkw). A-RDN induced significant increases in diuretic and natriuretic responses to GLP-1 in HF (urine flow 96.0 ± 1.9 vs. 53.4 ± 4.3 µl/min/gkw, sodium excretion 13.6 ± 1.4 vs. 7.4 ± 0.8 µEq/min/gkw).

**Conclusions:**

The excessive activation of neural circuitry involving afferent and efferent renal nerves suppresses diuretic and natriuretic responses to GLP-1 in HF. These pathophysiological responses to GLP-1 might be involved in the interaction between incretin-based medicines and established HF condition. RDN restores diuretic and natriuretic effects of GLP-1 and thus has potential beneficial therapeutic implication for diabetic HF patients.

## Background

Glucagon-like peptide-1 (GLP-1), incretin hormone, is secreted from epithelial cells in small intestine after meal ingestion and acts on the pancreas to promote insulin secretion. GLP-1 also suppresses appetite and increases energy expenditure to regulate glucose and energy metabolism [1, 2]. Based on these properties, GLP-1 related medicines are now widely used as one of the major strategies for treatment of type-2 diabetes and obesity. To date, cardiovascular outcome studies show that GLP-1 receptor (GLP-1R) agonists reduce atherosclerotic events including myocardial infarction and stroke, and also prevent progression of chronic kidney disease in type-2 diabetic patients [3–5]. On the other hand, GLP-1R agonists don’t affect the risk of heart failure (HF). Furthermore, the effects of GLP-1 on HF, especially established/symptomatic HF, are poorly understood and has been a subject of active debate [6–9].

GLP-1Rs are widely expressed in the whole body including the kidney [1, 10–15] and GLP-1 has diuretic and natriuretic effects in humans and animal models of hypertension, diabetes and obesity [10, 12, 13, 16]. As underlying mechanisms, it has been reported that GLP-1 causes renal vasodilatation, increases renal blood flow, increases glomerular filtration rate, inhibits the Na^+^/H^+^ exchanger 3 in proximal tubule and decreases sodium, bicarbonate and water reabsorption [12, 14]. In mice GLP-1R is expressed in the atrium of the heart and GLP-1R agonist promotes the secretion of atrial natriuretic peptide [17], which consequently causes diuresis and natriuresis. In addition to these direct effects on the kidney and the heart, it is likely that GLP-1 regulates diuresis and natriuresis via neural mechanisms as well. GLP-1 has been reported to act on neural pathways by acting on areas of the brain with a weak blood brain barrier [18–20] and vagal afferent [21–23]. Recently we have shown that GLP-1 activates afferent renal nerves to increase efferent renal sympathetic nerve activity that negates the diuresis and natriuresis produced by GLP-1 in normal rats [24]. However, the renal effects of GLP-1 in pathological conditions such as HF have not been elucidated to date.

The present study was conducted to assess the effect of GLP-1 on both the sensory afferent renal nerves and motor efferent renal sympathetic nerves in rats with HF, induced by coronary artery ligation and its relevance to diuresis and natriuresis, and hemodynamic changes in response to intravenous infusion of GLP-1. To address the relative contributions of afferent vs. efferent renal nerves to regulation of GLP-1 mediated diuresis and natriuresis in HF, either total renal denervation with surgical cutting of the renal nerves or a selective afferent renal denervation with periaxonal application of capsaicin were performed as demonstrated previously [24–26].

## Methods

### Animals

Male Sprague–Dawley rats weighing 220 to 250 g were purchased from Sasco Breeding Laboratories (Omaha, NE). Animals were housed with a 12-h light–dark cycle at ambient 22 °C 30–40% relative humidity. Laboratory chow and tap water were available ad libitum.

### Induction of heart failure

Rats were randomly assigned to either a Sham-operated control group or a HF group. HF was produced by left coronary artery ligation, as previously described with slight modification [27–30]. The rats were anesthetized with 3 to 4% isoflurane inhalation in an anesthesia chamber, and orally intubated to facilitate artificial ventilation. The tidal volume was adjusted for each animal to provide normal chest expansion. During the procedure, 2 to 2.5% isoflurane was used. Adequacy of the anesthesia was confirmed by the lack of the response to paw pinches during the surgical procedure. A left thoracotomy was performed at the 4th intercostal space. This allows visualization of the left coronary artery without having to lift the heart. In the HF group, the left coronary artery was ligated 1–2 mm below its origin from the aorta. The sham rats underwent thoracotomy and manipulation of the heart, but the coronary artery was not ligated. After these maneuvers, a silastic chest tube was inserted between the 5 and 6th ribs and the thorax was closed. The air within the thorax was aspirated by means of the chest tube while hyperinflating the lungs. The chest tube was then removed, and the rat was allowed to recover from the anesthesia and the endotracheal tube was removed. The rats were treated with analgesics (buprenorphine, 0.1 mg/kg, s.c.) for 3 postoperative days. The severity of HF was determined by both cardiac functional and histological assessment. A week before the terminal experiments, echocardiograms (Vevo 3100, 15-MHz probe; Visual Sonics, Inc., Toronto, Canada) were performed. Under anesthesia with 2 to 2.5% isoflurane inhalation, rats were placed on a heated stage that recorded electrocardiogram, respiratory rate and body temperature. Adequacy of the anesthesia was confirmed by the lack of the response to paw pinches and monitoring heart rate during the procedure. Left ventricular end-diastolic dimension (LVEDD), left ventricular end-systolic dimension (LVESD), fractional shortening (FS), ejection fraction (EF), left ventricular end-diastolic volume (LVEDV), left ventricular end-systolic volume (LVESV), stroke volume (SV) and cardiac output (CO) were analyzed by using Visual Sonics software. At the time of the terminal experiments, hemodynamic measurement was performed by using a Mikro-Tip catheter (SPR-407, Millar Instruments, Houston, TX) to obtain left ventricular end-diastolic pressure (LVEDP), left ventricular end-systolic pressure (LVESP), heart rate, maximal slope of systolic pressure increment (+dP/dt) and diastolic pressure decrement (−dP/dt). Further, to anatomically assess the extent of HF, infarct size of the left ventricle (LV) was calculated. The heart was dissected and the atria and right ventricle were removed. The LV was longitudinally cut and laid flat. A digital image of the LV was captured by a digital camera. Infarct size was measured by dividing the size of the infarcted area by the total size of the LV using ImageJ software (National Institutes of Health). Rats with both LVEDP ˃ 15 mmHg and infarct size ˃ 30% were considered to be in HF. All experiments were performed 5–6 weeks after coronary ligation surgery, unless otherwise specifically noted.

### Intrapelvic injection of GLP-1 with direct recording of afferent renal nerve activity (ARNA)

Rats were anesthetized with urethane (0.75 g/kg, i.p.) and α-chloralose (70 mg/kg, i.p.). This anesthetic is specifically chosen to minimize the effects of anesthesia on central pathways involved in autonomic regulation of the cardiovascular system. Under this anesthesia a lot of the autonomic reflex functions are studied and are operational [24, 26, 29, 31–33] and therefore being used in current study examining effects of GLP-1 on neural circuitry of renal nerve activity involved in central nervous system. Body temperature was controlled at 36–38 °C by a stage heater. Tracheal intubation was performed to make the rats breathe independently. The PE-50 polyethylene tubing was insert into the right femoral artery and connected to a pressure transducer. PowerLab software (8SP, AD Instruments, Colorado Spring, CO) was used to record mean arterial pressure (MAP) and heart rate (HR), simultaneously. The right femoral vein was cannulated with PE-50 tubing for intravenous supplemental anesthesia. Adequacy of the anesthesia was confirmed by the lack of the response to paw pinches during the surgical procedure. The direct recording of ARNA with intrapelvic injections was performed as described previously [24, 26, 34, 35]. Briefly, the left kidney was exposed through a retroperitoneal flank incision. The renal pelvis was cannulated with a 32 G triple-lumen catheter (ReCathCo, Allison Park, PA) via the ureter for intrapelvic perfusion and withdrawal of infusate and monitoring of pelvic pressure. A renal nerve branch located at the renal hilus was isolated and gently put on a bipolar silver electrode. The nerve-electrode junction was electrically isolated from surrounding tissue with gel (Wacker, St. Louis, MO). Grass amplifier was used to amplify the electrical signal with high- and low-frequency cutoffs of 1000 Hz and 100 Hz, respectively. The PowerLab system was used to record the rectified output (resister capacitor filtered, time constant, 0.5 s) and integrate the raw nerve discharge. First, the total basal renal nerve discharge was recorded. Then, the afferent nerve discharge and background noise were obtained after sectioning proximal end of the nerve to eliminate efferent nerve signal. Finally, the background noise was recorded after sectioning the distal end of the nerve. The value of ARNA was obtained by subtracting the background noise from the integrated signal. For description of ARNA response to intrapelvic injection of GLP-1 (3 μM in saline, Enzo Life science, Farmingdale, NY), ARNA was expressed as a percent changes from the baseline value and also normalized to maximal ARNA induced by intrapelvic injection of capsaicin (100 μM in 5% ethanol and 95% saline, Sigma, Saint Louis, MO) thus expressed as a percent of ARNAmax as shown previously [24, 26, 34]. At the end of the experiment, rats were euthanized by an overdose of pentobarbital (150 mg/kg, i.p.).

### Real-time quantitative reverse-transcription polymerase chain reaction (qRT-PCR) for GLP-1R in the renal pelvis

The kidneys of rats were removed and frozen on dry ice and then stored at − 80 °C. Total RNA from pelvis in the kidney was isolated using TRIzol (Invitrogen, Madison, WI) and the purity and concentration of total RNA was quantified using Nanodrop 2000C (Thermo Fisher Scientific, Waltham, MA). Pure quality (A260/A280 > 1.8–2.0) RNA was used for cDNA synthesis and qPCR assay. First-strand cDNA synthesis was completed from 1 µg of RNA using the iScript cDNA Synthesis Kit (catalog number: 170-8841, Bio-Rad Laboratories, Hercules, CA). The qPCR assay was performed using 2 × iTaq Universal SYBR Green Supermix (catalog number: 1725121, Bio-Rad Laboratories, Hercules, CA) according to the Bio-Rad manufacturer’s instructions. The qPCR mixture was amplified with the protocol 95 °C for 5 min pre-denaturation and then 35 cycles of 95 °C for 20 s, 57 °C for 30 s, and 72 °C for 45 s in a Bio-Rad CFX qPCR System. A 10 µl qPCR reaction was amplified in duplicate wells for each sample 4.5 µl of cDNA mixture (100 ng cDNA template + rest nuclease-free water) with 5 µl iTaq Universal SYBR Green Supermix, and 0.5 µl forward and reverse primer mixtures (10 pm). Bio-Rad CFX Manager 3.0 software was used for the relative quantification of mRNA expression in fold change (2^−ΔΔCt^) using 18sRNA as a reference control. The details of primer sequences used are: Rat GLP-1R forward; 5′-CTTTGATGACTACGCCTGCT-3′, reverse; 5′-CTTGGACTCTTCGCACTCC-3′ and, rat 18S ribosomal RNA: forward; 5′-TGTGATGCCCTTAGATGTCC-3′ reverse; 5′-TTATGACCCGCACTTACTGG-3′.

### Western blot analysis for protein expression of GLP-1R in the renal pelvis

The kidneys of rats were removed, frozen on dry ice and then stored at − 80 °C. The protein extracts from pelvis in the kidney were used for western blot analysis as described previously [24, 26]. Samples were processed for protein isolation using RIPA buffer (Boston Bio-product, Ashland, MA) with protease inhibitors cocktail (Cell signaling technology, Danvers, MA), quantified by Pierce BCA Protein Assay Kit (Thermo Fisher Scientific, Waltham, MA) and 40 μg of proteins were boiled with 4× denaturing Laemmli Sample Buffer (Bio-Rad Laboratories, Hercules, CA) and loaded on 7.5% SDS-PAGE gel. Post-electrophoresis, gels were transferred onto polyvinylidene difluoride membranes, which were then blocked with 5% non-fat dried milk in tris-buffered saline with tween 20 (TBST) for 1 h at room temperature. The membranes were incubated with primary antibody (mouse anti-GLP-1R, sc-390774, Santa Cruz Biotechnology, Santa Cruz, CA, 1:1000 and rabbit anti-GAPDH, sc-25778, Santa Cruz Biotechnology, Santa Cruz, CA, 1:1000) in TBST overnight at 4 °C. After washing, the membranes were incubated with secondary antibodies conjugated with horseradish peroxidase diluted at 1:5000 in TBST for 1 h at room temperature. Signals for protein bands were developed with Super Signal West Femto Chemiluminescent kit (Thermo Fisher Scientific, Waltham, MA) and detected by a Molecular Imager ChemiDoc XRS imaging system (Bio-Rad Laboratories, Hercules, CA). The protein bands were quantified by Image Lab software version 6 (Bio-Rad Laboratories, Hercules, CA). The protein levels of GLP-1R were expressed as the intensity of its band signal normalized to the intensity of the GAPDH signal.

### Intravenous infusion of GLP-1 with direct recording of renal sympathetic nerve activity (RSNA)

Rats were anesthetized with urethane (0.75 g/kg, i.p.) and α-chloralose (70 mg/kg, i.p.). This anesthetic is chosen due to specific reasons described in the direct recording of ARNA. Adequacy of the anesthesia was confirmed by the lack of the response to paw pinches during the surgical procedure. The PE-50 polyethylene tubing was inserted into the right femoral artery and connected to a pressure transducer. PowerLab software (8SP, AD Instruments, Colorado Spring, CO) was used to record MAP and HR, simultaneously. Another PE-50 tubing was inserted into the right femoral vein to infuse isotonic saline (0.9% NaCl) at a rate of 50 µl/min throughout the experiment, except the period of GLP-1 infusion (1 µg/kg/min) for 30 min. RSNA recording was performed as described in ARNA recording without cutting the renal nerve. The background noise was recorded after cutting both the proximal and the distal end of the nerve. The peak change in RSNA response to intravenous infusion of GLP-1 was expressed as a percent of the baseline level as shown previously [24]. At the end of the experiment, rats were euthanized by an overdose of pentobarbital (150 mg/kg, i.p.).

### Acute total renal denervation (T-RDN)

Acute T-RDN was performed as described previously [30, 31, 36]. Briefly, the hilus of the left kidney was exposed retroperitoneally and all visible renal nerves were cut by using microsurgical scissors. Then 95% ethanol was painted around the renal artery and vein by using cotton swabs to ensure ablation of remaining nerve fibers. This technique has previously been validated as the efficacy in reducing renal norepinephrine content to under 5% of the normal levels [30, 31, 36].

### Chronic selective afferent renal denervation (A-RDN)

Four weeks after coronary artery ligation surgery, afferent renal denervation was performed as described previously [24–26]. Briefly, rats were anesthetized with 2 to 2.5% isoflurane inhalation. Adequacy of the anesthesia was confirmed by the lack of the response to paw pinches during the surgical procedure. The kidneys were exposed through abdominal midline incision. A small piece of filter paper soaked in a capsaicin solution (33 mM in 10% ethanol, 10% tween80 and 80% saline, Sigma, Saint Louis, MO) was wrapped around the vessels for 10 min. During the procedure, parafilm was placed under the vessels to prevent capsaicin exposure to any other adjacent visceral tissues. After 10 min of capsaicin exposure, the filter paper and parafilm were removed. The same procedure was conducted on the contralateral side. The rats were treated with analgesics (buprenorphine, 0.1 mg/kg, s.c.) for 3 postoperative days. The acute terminal experiments were performed at between 1 and 2 weeks after A-RDN (5–6 weeks after the coronary artery ligation). To validate the effectiveness of A-RDN, protein levels of calcitonin gene-related peptide (CGRP), which is the dominant neurotransmitter for afferent renal nerves [37, 38], and tyrosine hydroxylase (TH), a marker for efferent sympathetic fibers in the renal pelvis were quantified. The western blot analysis for CGRP (rabbit anti-CGRP, BML-CA1134, Enzo Life Sciences, Farmingdale, NY, 1:2000) and TH (mouse anti-TH, sc-25269, Santa Cruz Biotechnology, Santa Cruz, CA, 1:1000) on protein extracts from renal pelvis were performed as described above with 12% SDS-PAGE gel to quantify the extent of ablation of afferent sensory nerves and determine if there was any damage to the efferent adrenergic innervation due to selective A-RDN in the pelvic wall of the kidney.

### Measurement of hemodynamic, diuretic and natriuretic responses to GLP-1

Rats were assigned randomly to one of six groups: Sham, Sham + T-RDN, Sham + A-RDN, HF, HF + T-RDN, HF + A-RDN with n = 6–10/group. Rats were anesthetized with Inactin (100 mg/kg, i.p.). This anesthetic is specifically chosen to minimize the effects on changes in blood pressure and thus provide an ideal perfusion pressure to the kidneys to perform renal function study. Under this anesthesia a lot of renal function studies [24, 28, 30, 31, 39, 40] are operational and therefore being used in current study examining effects of GLP-1 on renal hemodynamics and excretion. Body temperature was controlled at 36–38 °C by a stage heater. Tracheal intubation was performed to make the rats breathe independently. The PE-50 tubing was inserted into the right femoral artery and connected to a pressure transducer for monitoring of MAP and HR. Another PE-50 tubing was inserted into the right femoral vein for isotonic saline infusion (50 µl/min) to create steady state condition for urine production and for GLP-1 infusion (1 µg/kg/min). For collecting urine, both ureters were approached retroperitoneally and cannulated with PE-10 tubing individually. The surgical procedures were completed within an hour. Adequacy of the anesthesia was confirmed by the lack of the response to paw pinches during the surgical procedure. Two urine collections (15 min each) were obtained over 30 min baseline period before starting GLP-1 infusion (1 µg/kg/min, i.v.). During GLP-1 infusion, urine was collected at 10, 20 and 30 min. After the termination of GLP-1 infusion, urine was collected at 40, 50 and 60 min. Urine volume was measured by subtracting the weight of tube before collecting urine from the weight of tube containing urine. A selective ion electrode (Beckman ion analyzer, Brea, CA) was used to measure sodium concentration of the urine samples. The creatinine levels in the urine and blood samples collected in citrate-containing tube were measured by using commercially available assay kits (ab65340, Abcam, Cambridge, MA). Creatinine clearance (CrCl) was then calculated. At the end of the experiments, rats were euthanized by an overdose of pentobarbital (150 mg/kg, i.p.) and then heart and kidneys were harvested.

### Statistical analysis

All data are expressed as mean ± SEM. Data were tested for significance using a Student’s *t*-test for two normally distributed groups. Data from three groups or more were assessed by an ordinary one-way ANOVA test followed by Bonferroni post hoc analysis. Data from experiments with two or more variables were assessed by two-way ANOVA followed by Bonferroni post hoc analysis. *P*-values < 0.05 were indicative of statistical significance. All statistical analysis was performed using GraphPad Prism 7.0.

## Results

### Cardiac characteristics of the HF Model

The baseline cardiac characteristics of Sham and HF rats (5–6 weeks after coronary artery ligation) used in this study are summarized in Table 1. The infarcted area in HF group was approximately 40% of the endocardial surface of the left ventricle. Sham rats had no observable damage to the myocardium. LVEDP was significantly increased in HF group compared to Sham group. The +dP/dt and −dP/dt were significantly decreased in HF group compared with Sham group, indicating decreased contractility and relaxation of the left ventricle in HF group. LVEDD, LVESD, LVEDV and LVESV were significantly increased in HF group compared to Sham group. FS, EF, SV and CO were greatly reduced in HF group compared to Sham group. The cardiac characteristics observed in this study are consistent with previous reports using this model of HF [41–43]. Additionally, substantial number of previous studies [44–51] demonstrated that natriuretic peptide levels in both the heart and the serum are significantly increased in rats with HF induced by coronary artery ligation used in the current study. Taken together, the ˃ 30% infarct size, increased LVEDP, LVEDD, LVESD, LVEDV and LVESV and decreased dP/dt, FS, EF, SV and CO all indicate that rats in HF group were experiencing cardiac dysfunction.

**Table 1:** Baseline cardiac characteristics of Sham and HF rats

	Sham (n = 10)	HF (n = 10)
Body weight: BW (g)	356 ± 6	370 ± 7
Heart weight: HW (g)	1.2 ± 0.1	1.6 ± 0.1*
HW/BW * 1000	3.2 ± 0.1	4.3 ± 0.2*
Infarct size (% of endocardial LV)	0	39 ± 2*
LVEDP (mmHg)	3 ± 1	21 ± 3*
LVESP (mmHg)	119 ± 4	106 ± 3
Heart rate (bpm)	350 ± 14	361 ± 14
+dP/dt (mmHg/s)	7161 ± 584	4783 ± 281*
−dP/dt (mmHg/s)	− 6878 ± 552	− 3745 ± 172*
LVEDD (mm)	8.0 ± 0.2	10.4 ± 0.2*
LVESD (mm)	4.8 ± 0.1	8.5 ± 0.2*
Fractional shortening (%)	39 ± 1	18 ± 1*
Ejection fraction (%)	64 ± 1	33 ± 1*
LVEDV (μl)	428 ± 27	653 ± 45*
LVESV (μl)	151 ± 14	443 ± 39*
Stroke volume (μl)	277 ± 14	209 ± 10*
Cardiac output (μl/min)	105 ± 2	66 ± 3*

Values are mean ± SEM; n = 10 for each group of rats. LVEDP, left ventricular end-diastolic pressure

*LVESP* left ventricular end-systolic pressure, *+dP/dt* maximal slope of systolic pressure increment. *−dP/dt* maximal slope of diastolic pressure decrement, *LVEDD* left ventricular end-diastolic dimension, *LVESD* left ventricular end-systolic dimension, *LVEDV* left ventricular end-diastolic volume, *LVESD* left ventricular end-systolic volume

*P < 0.05 compared to Sham

### Intrapelvic injection of GLP-1 increases ARNA

**Fig. 1:**
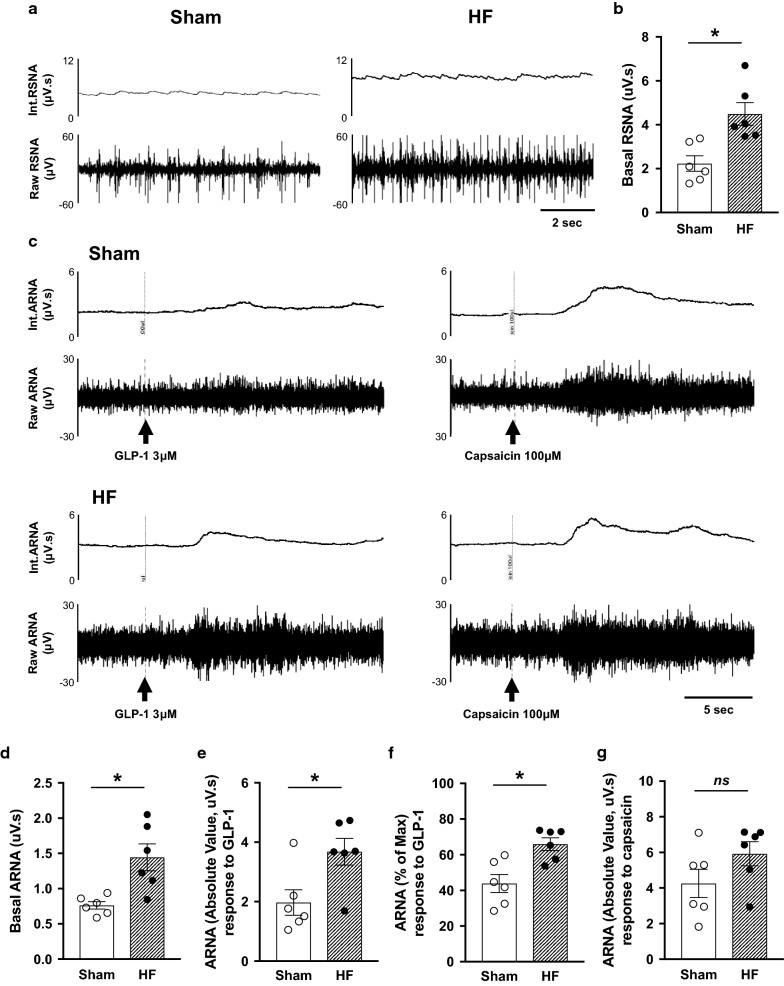
ARNA response to intrapelvic injection of GLP-1. **a** Raw tracings of integrated and RSNA from Sham and HF rats. **b** Summary data for basal RSNA. **c** Raw tracings of integrated and ARNA from Sham and HF rats. The ARNA was recorded before and after intrapelvic injection of GLP-1 3 μM or capsaicin 100 μM to determine the peak level of ARNA (ARNAmax). **d** Summary data for ARNA. **e** Summary data for peak ARNA responses to GLP-1 expressed as an absolute value. **f** Summary data for peak ARNA responses to GLP-1 expressed a percentage of ARNAmax. **g** Summary data for peak ARNA responses to capsaicin. n = 6. *P < 0.05 vs. Sham

Direct recordings of ARNA responses to intrapelvic injection of GLP-1 and capsaicin from Sham and HF rats are shown in Fig. 1. The basal total RSNA was significantly higher in HF rats compared to Sham rats (4.49 ± 0.52 vs. 2.23 ± 0.36 µV s, *P *< 0.05, Fig. 1a, b). The basal ARNA after sectioning the proximal end of the renal nerve was higher in HF rats (1.44 ± 0.16 vs. 0.76 ± 0.05 µV s, *P *< 0.05, Fig. 1c, d), consistent with our previous report [25]. Intrapelvic injection of GLP-1 increased ipsilateral ARNA in both Sham and HF rats (Fig. 1c). The peak response to intrapelvic GLP-1 was higher in HF rats (3.68 ± 0.38 vs. 1.97 ± 0.39 µV s, P < 0.05, 66.0 ± 3.0 vs 43.9 ± 4.6% of ARNAmax, *P *< 0.05, Fig. 1e, f). The peak response to intrapelvic capsaicin to elicit maximal activation was not statistically different between Sham and HF rats (4.26 ± 0.73 vs. 5.92 ± 0.57 µV s, *P *> 0.05, Fig. 1c, g). These results demonstrated that not only the resting levels but also the GLP-1-induced activation levels of ARNA were greater in HF compared to Sham.

### GLP-1R expression in the renal pelvis

**Fig. 2:**
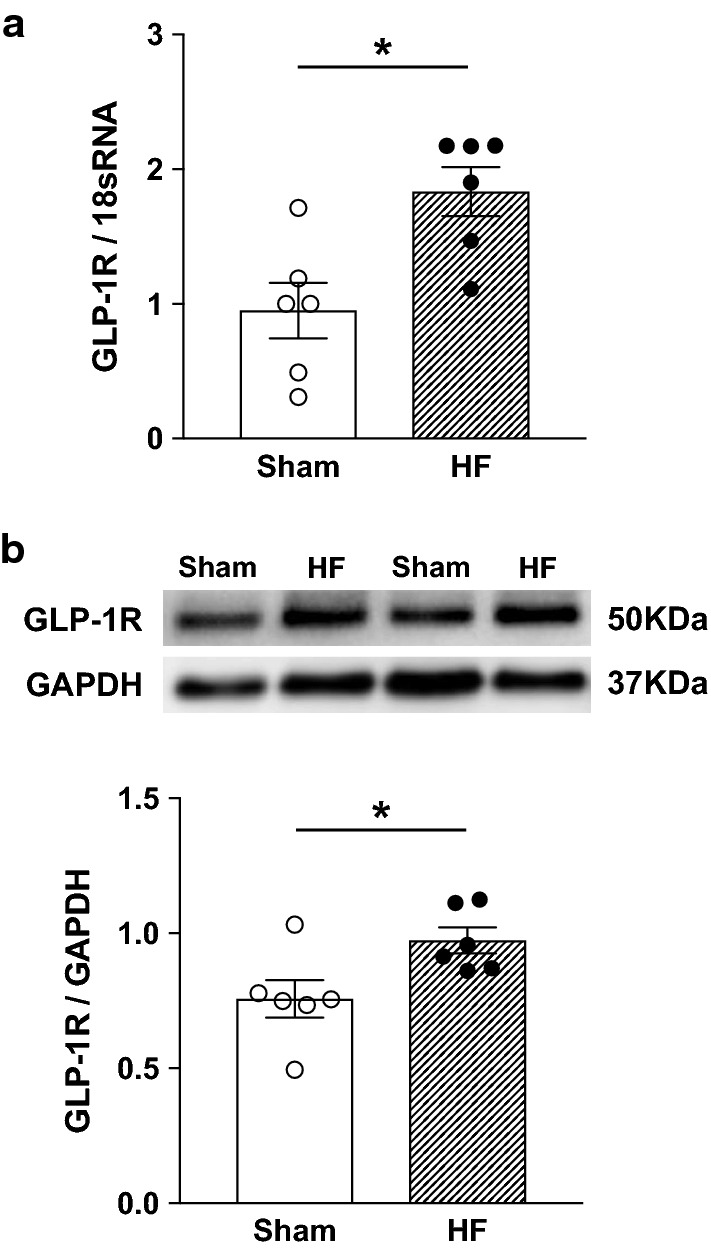
GLP-1R expression in the renal pelvis. Real-time qRT-PCR (**a**) and western blot analysis (**b**) for GLP-1R in the renal pelvis of Sham and HF rats. n = 6. *P < 0.05 vs. Sham

Real-time qRT-PCR and western blot analysis revealed that there were significant increases in both mRNA (Fig. 2a) and protein (Fig. 2b) levels of GLP-1R in the renal pelvis of rats with HF compared to Sham.

### Intravenous infusion of GLP-1 increases RSNA, MAP and HR

**Fig. 3:**
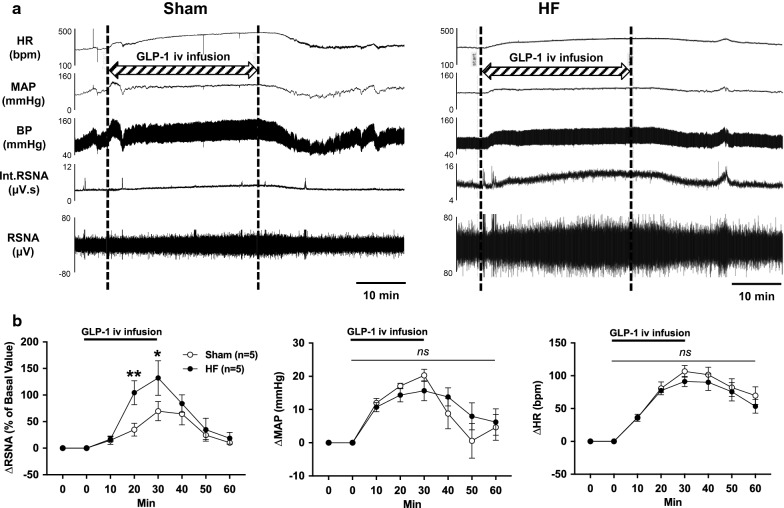
RSNA, MAP and HR responses to intravenous infusion of GLP-1. **a** Raw tracings of changes in RSNA, MAP and HR to intravenous (i.v.) infusion of GLP-1 in Sham and HF rats. **b** Summary data for the effect of GLP-1 on changes in RSNA, MAP and HR over 60 min. n = 5. *P < 0.05, **P < 0.01 vs. Sham

Direct recordings of RSNA, MAP and HR responses to intravenous infusion of GLP-1 from Sham and HF rat are shown in Fig. 3. Intravenous infusion of GLP-1 increased RSNA, MAP and HR in both Sham and HF rats. The increase in RSNA response to intravenous infusion of GLP-1 was higher in HF rats (132.1 ± 29.2% vs. 69.8 ± 16.1% of basal value, *P *< 0.05, Fig. 3b). The increases in MAP and HR responses to intravenous infusion of GLP-1 were not significantly different between Sham and HF rats (Fig. 3b). These results demonstrated that not only the resting levels but also the GLP-1-induced activation levels of total RSNA was greater in HF compared to Sham.

### Intravenous infusion of GLP-1 induces diuresis and natriuresis

**Fig. 4:**
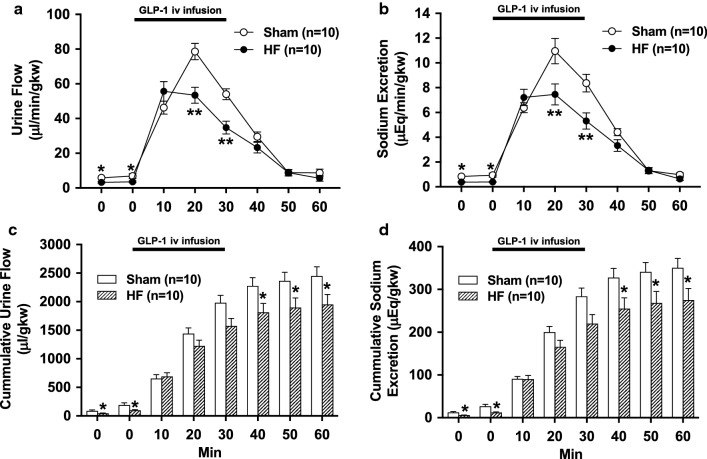
Diuretic and natriuretic responses to intravenous infusion of GLP-1. Urine flow (**a**) and sodium excretion (**b**) in response to GLP-1 injection in Sham and HF rats. Cumulative values of urine flow (**c**) and sodium excretion (**d**) in response to GLP-1 injection in Sham and HF rats. n = 10. *P < 0.05, **P < 0.01 vs. Sham

Intravenous infusion of GLP-1 increased urine flow and sodium excretion in both Sham and HF rats. Diuretic and natriuretic responses to GLP-1 were blunted in HF rats (urine flow at 20 min 53.4 ± 4.3 vs. 78.6 ± 4.4 µl/min/gkw, sodium excretion at 20 min 7.4 ± 0.8 vs. 10.9 ± 1.0 µEq/min/gkw, *P *< 0.01, n = 10, Fig. 4a, b). Baseline levels of cumulative urine flow and sodium excretion were decreased in HF rats (98.5 ± 12.6 vs. 191.1 ± 36.4 µl/gkw and 12.3 ± 1.1 vs. 26.8 ± 4.2 µEq/gkw, respectively, *P *< 0.05, n = 10, Fig. 4c, d). Cumulative urine flow and sodium excretion responses to GLP-1 were also decreased in HF rats (1951 ± 166 vs. 2451 ± 151 µl/gkw and 275 ± 25 vs. 351 ± 21 µEq/gkw at 60 min, respectively, *P *< 0.05, n = 10, Fig. 4c, d).

### Acute T-RDN enhances diuretic and natriuretic responses to GLP-1

**Fig. 5:**
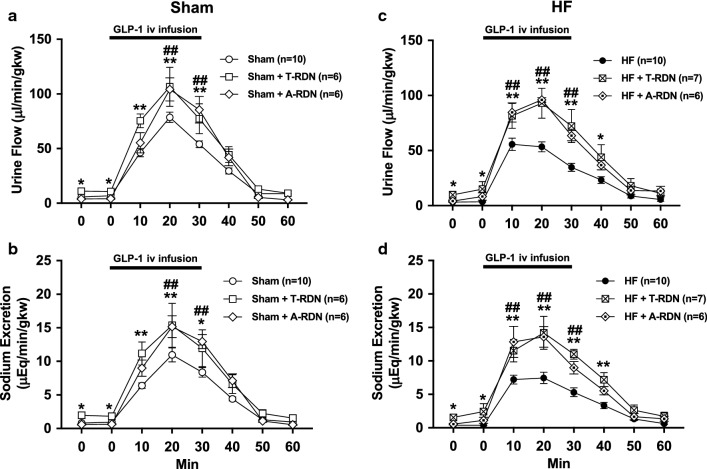
Effects of T-RDN or A-RDN on diuretic and natriuretic responses to intravenous infusion of GLP-1. Urine flow (**a**, **c**) and sodium excretion (**b**, **d**) in response to GLP-1 injection in Sham (**a**, **b**) and HF (**c**, **d**) rats with or without T-RDN and A-RDN. n = 6–10. *P < 0.05, **P < 0.01 between rats with and without T-RDN. ^#^*P *< 0.05, ^##^*P *< 0.01 between rats with and without A-RDN

Acute T-RDN was performed by surgical sectioning followed by perivascular application of 95% ethanol on the around the renal artery and vein. The data of urine flow and sodium excretion from left denervated kidneys in Sham + T-RDN and HF + T-RDN groups were compared with that from intact kidneys in Sham and HF groups, respectively. T-RDN increased baseline levels of cumulative urine flow and sodium excretion in Sham + T-RDN group (326.4 ± 61.5 vs. 191.1 ± 36.4 µl/gkw and 57.0 ± 13.2 vs. 26.8 ± 4.2 µEq/gkw, respectively, *P *< 0.05, n = 6–10) and HF + T-RDN group (374.6 ± 134.2 vs. 98.5 ± 12.6 µl/gkw and 59.4 ± 15.7 vs. 12.3 ± 1.1 µEq/gkw, respectively, *P *< 0.05, n = 7–10). T-RDN enhanced diuretic and natriuretic responses to GLP-1 in Sham + T-RDN group (urine flow at 20 min 106.5 ± 16.2 vs. 78.6 ± 4.4 µl/min/gkw, sodium excretion at 20 min 15.4 ± 3.0 vs. 10.9 ± 1.0 µEq/min/gkw, *P *< 0.01, n = 6–10, Fig. 5a, b) and HF + T-RDN group (urine flow at 20 min 93.0 ± 12.6 vs. 53.4 ± 4.3 µl/min/gkw, sodium excretion at 20 min 14.2 ± 1.6 vs. 7.4 ± 0.8 µEq/min/gkw, *P *< 0.01, n = 7–10, Fig. 5c, d).

### Chronic A-RDN enhances diuretic and natriuretic responses to GLP-1

**Fig. 6:**
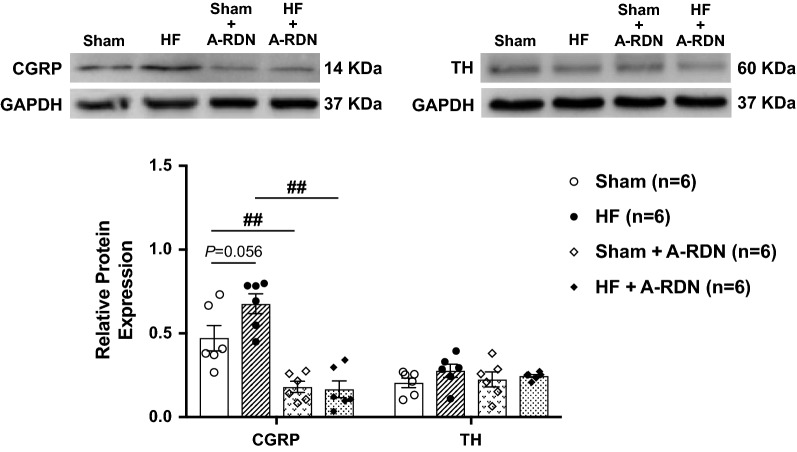
Validation of A-RDN by western blot analysis. Relative CGRP and TH protein expression in the renal pelvis of Sham and HF rats with or without A-RDN. n = 6. ^##^*P *< 0.01 between rats with and without A-RDN

A-RDN was performed by bilateral perivascular application of capsaicin on the renal nerves. Either 1 or 2 weeks after A-RDN, there were enhanced diuretic and natriuretic responses to GLP-1 in Sham + A-RDN group (urine flow at 20 min 104.2 ± 9.6 vs. 78.6 ± 4.4 µl/min/gkw, sodium excretion at 20 min 15.4 ± 3.0 vs. 10.9 ± 1.0 µEq/min/gkw, *P *< 0.01, n = 6–10, Fig. 5a, b) and HF + A-RDN group (urine flow at 20 min 96.0 ± 1.9 vs. 53.4 ± 4.3 µl/min/gkw, sodium excretion at 20 min 13.6 ± 1.4 vs. 7.4 ± 0.8 µEq/min/gkw, *P *< 0.01, n = 6–10, Fig. 5c, d). The effects of T-RDN on responses of increases in urine flow and sodium excretion to GLP-1 were not significantly different from those observed with A-RDN. Figure 6 shows the protein expression of CGRP and TH in the renal pelvis of rats with or without capsaicin treatment. CGRP was decreased by 62–75% in rats with capsaicin treatment, while TH was not different between the rats with and without capsaicin treatment. These results indicate that capsaicin treatment selectively ablated renal afferent innervation but did not affect renal efferent noradrenergic innervation, validating the effectiveness of capsaicin treatment to produce A-RDN.

### Either T-RDN or A-RDN attenuates increases in MAP and HR responses to GLP-1

**Fig. 7:**
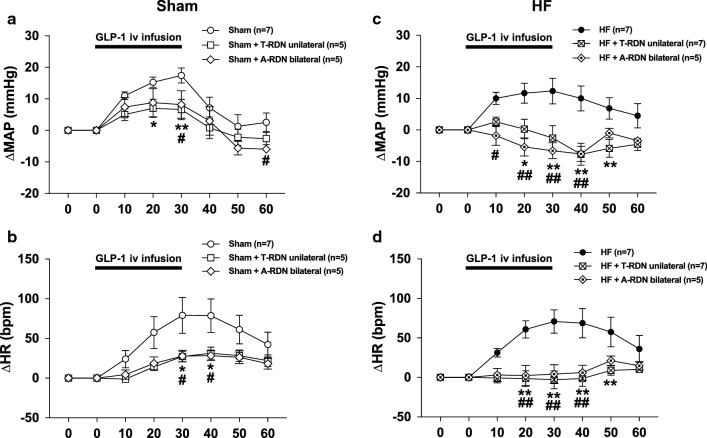
Effects of T-RDN or A-RDN on MAP and HR responses to intravenous infusion of GLP-1. MAP and HR changes in response to GLP-1 injection in Sham (**a**, **b**) and HF (**c**, **d**) rats with or without T-RDN and A-RDN. n = 5–7. *P < 0.05, **P < 0.01 between rats with and without T-RDN. ^#^*P *< 0.05, ^##^*P *< 0.01 between rats with and without A-RDN

MAP and HR were significantly increased during intravenous infusion of GLP-1 in both Sham and HF rats. There were no statistical differences in MAP and HR between Sham and HF rats. These changes in MAP and HR to GLP-1 were significantly attenuated by either T-RDN or A-RDN in both Sham and HF rats. These changes in MAP and HR to GLP-1 were not statistically different between the two groups, Sham + T-RDN and Sham + A-RDN (Fig. 7a, b) or HF + T-RDN and HF + A-RDN (Fig. 7c, d). Table 2 shows the changes of CrCl during intravenous infusion of GLP-1. Baseline CrCl was lower in HF than Sham (817 ± 101 vs. 1356 ± 109 µl/min/gkw, *P *< 0.05, n = 5). T-RDN increased baseline CrCl in both Sham + T-RDN (1893 ± 137 vs. 1356 ± 109 µl/min/gkw, *P *< 0.05, n = 5) and HF + T-RDN group (1858 ± 215 vs. 817 ± 101 µl/min/gkw, *P *< 0.05, n = 5). Intravenous infusion of GLP-1 significantly increased CrCl in both Sham + T-RDN (2978 ± 213 vs. 2225 ± 94 µl/min/gkw, *P *< 0.05, n = 5) and HF + T-RDN group (2868 ± 178 vs. 1725 ± 108 µl/min/gkw, *P *< 0.05, n = 5). CrCl response to GLP-1 in HF + T-RDN was comparable to that in Sham + T-RDN.

**Table 2:** Changes of CrCl during intravenous infusion of GLP-1

	Sham (n = 5)	HF (n = 5)	Sham + T-RDN (n = 5)	HF + T-RDN (n = 5)
CrCl (µl/min/gkw)				
Baseline	1356 ± 109	817 ± 101^†^	1893 ± 137^‡^	1858 ± 215^‡^
20 min	2225 ± 94*	1725 ± 108*^†^	2978 ± 213*^‡^	2868 ± 178*^‡^

Values are mean ± SEM; n = 5 for each group of rats

*CrCl* creatinine clearance

*P < 0.05 compared to baseline. ^†^P < 0.05 compared between Sham and HF. ^‡^P < 0.05 compared between the group with and without T-RDN

## Discussion

We have shown that baseline ARNA was elevated in rats with HF. The response of an increase in ARNA to intrapelvic injection of GLP-1 was enhanced in HF. Consistent with these observations GLP-1R expression in the renal pelvis was augmented in HF. The response of an increase in RSNA to intravenous infusion of GLP-1 was also exaggerated in HF. Diuretic and natriuretic responses to GLP-1 were blunted in HF and restored by either T-RDN or A-RDN to the comparable levels with that in Sham. These changes to GLP-1 were not significantly different between T-RDN and A-RDN in both HF and Sham groups. The main findings deduced by the results in this study are as follow: (1) GLP-1 increases RSNA to regulate diuresis and natriuresis in an inhibitory manner, in which the afferent renal nerve activation is potentiated via elevated GLP-1R expression in the renal pelvis of rats with HF. (2) Either T-RDN or A-RDN inhibits the activation of neural circuitry utilizing the renal nerves to enhance the diuretic and natriuretic responses to GLP-1.

We have shown that basal ARNA was higher in HF than Sham consistent with our previous report [26] as well as basal RSNA [29, 32, 33]. Intrapelvic injection of GLP-1 increased ARNA and this response was 1.5-fold greater in HF compared to Sham. One possible mechanism by which there would be enhanced response to GLP-1 in HF rats is that there is an altered expression of the GLP-1R within the renal pelvis of rats with HF. Thus, we investigated GLP-1R expressions in the renal pelvis of rats with HF by real-time qRT-PCR and western blot analysis. The mRNA levels of GLP-1Rs in the pelvis were increased in HF compared to Sham. Regarding western blot analysis, it has been reported that conventional polyclonal antibodies against the GLP-1R exhibit suboptimal sensitivity and lack of specificity [11, 13, 14, 52]. In current study, we used newly developed monoclonal antibody that has previously been validated as specific for GLP-1R [24] and demonstrated that the protein levels of GLP-1Rs in the pelvis are greater in HF than Sham, suggesting that enhanced expression of GLP-1R in the pelvis leads to enhanced activation of afferent renal nerves in rats with HF. On the other hand, a previous report shows that the plasma level of active GLP-1 is not changed in rats with HF induced by coronary artery ligation compared to Sham [53]. Our and this finding imply that enhanced activation of afferent renal nerves by GLP-1 in HF is dependent on the expression levels of GLP-1R rather than the concentrations of GLP-1 itself in the renal pelvis.

**Fig. 8:**
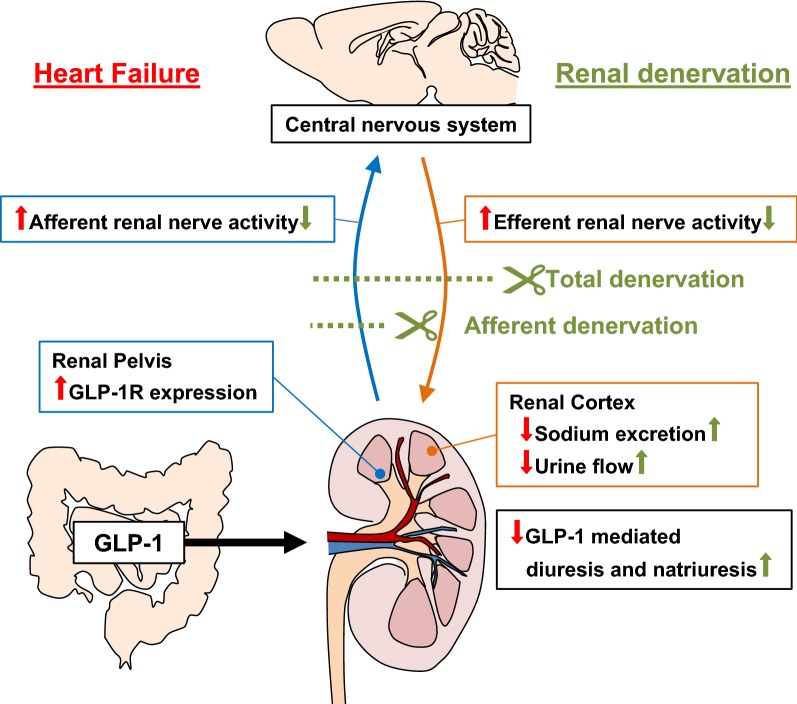
Proposed model for neural mechanisms in regulating GLP-1 mediated diuresis and natriuresis in HF. GLP-1 activates afferent renal nerve via GLP-1R expressed in the renal pelvis to increase efferent renal nerve activity that regulate diuresis and natriuresis in an inhibitory manner. In HF condition, there is an excessive activation of afferent renal nerve due to an overexpression of GLP-1R in the renal pelvis resulting in an excessive activation of efferent renal nerve thereby suppressing diuresis and natriuresis. Either A-RDN or T-RDN inhibits the activation of neural circuitry utilizing the renal nerves to restore GLP-1 mediated diuresis and natriuresis

Further, increase in RSNA by intravenous infusion of GLP-1 was 1.9-fold higher in HF than Sham, while increases in MAP and HR responses to GLP-1 were not significantly different between HF and Sham. It is well known that activation of efferent renal nerves exhibits the anti-diuretic effect by increased renin secretion, sodium reabsorption and renal vascular resistance [37, 38]. Hence, it is plausible that in HF greater increase in RSNA attenuates diuretic and natriuretic responses to GLP-1. As underling mechanism of the interaction between afferent and efferent renal nerves our previous report [24] has shown that activation of afferent renal nerves leads to activation of efferent renal sympathetic nerves response to GLP-1, possibly via central neural reflex in normal rats. In this study we demonstrated that intravenous infusion of GLP-1 increased total RSNA, ARNA and efferent renal nerve activity (ERNA). It is notable that ERNA recording obtained by cutting the distal end of the renal nerve to eliminate the afferent signal, demonstrated that response of increase in ERNA to GLP-1 is significantly diminished compared to that in total RSNA with intact renal nerves. These findings suggest that an increase in ARNA elicits the increase in ERNA to augment total RSNA. This hypothesis is further supported by a study [54] which demonstrated that electrical stimulation of afferent renal nerves activates rostral ventrolateral medulla-projecting paraventricular nucleus (PVN) neurons in the hypothalamus. These pre-autonomic neurons in the PVN are known to regulate sympathetic outflow to various peripheral organs including the kidney. Taken together, we propose that in HF enhanced activation of afferent renal nerves leads to enhanced activation of efferent renal sympathetic nerves response to GLP-1 possibly via central nervous system, thereby suppressing diuresis and natriuresis (Fig. 8).

There have been several previous studies addressing changes in diuretic and natriuretic responses to GLP-1 in disease conditions [55–57]. Some of these studies suggest that GLP-1-induced diuresis and natriuresis are attenuated in spontaneously hypertensive rats compared to normotensive rats [56, 57]. These studies demonstrate that there is none or very little GLP-1R expression in the renal vasculature and GLP-1-induced renal artery vasodilation leading to increases in renal blood flow and glomerular filtration rate is diminished in spontaneously hypertensive rats [56, 57]. On the other hand, another study suggests that natriuretic effect of GLP-1R agonist excendin-4 is preserved in diabetic db/db mice [55]. Our current study is first to demonstrate that GLP-1-induced diuresis and natriuresis are attenuated in rats with HF induced by coronary artery ligation, in which the excessive activation of neural circuitry involving afferent and efferent renal nerves by GLP-1 might be implicated. These neural effects of GLP-1 possibly interact with the gut-renal axis regulating postprandial sodium and water homeostasis. The dysregulation of these neural mechanisms might contribute to renal sodium and fluid retention associated with HF.

In current study, T-RDN by itself caused significantly greater urine flow, sodium excretion and increased CrCl at baseline in both HF and Sham. These results are consistent with previous studies [30, 39]. The extent of increases in diuretic and natriuretic, as well as CrCl responses to GLP-1 by T-RDN was greater in HF than Sham. These data suggest that in the kidneys with intact nerves, the anti-diuretic effect and inhibition of increase in CrCl induced by activation of efferent renal nerves response to GLP-1 are greater in HF than Sham. T-RDN inhibited GLP-1 mediated activation of efferent renal sympathetic nerve, thereby eliciting an enhanced diuresis and natriuresis, and increases in CrCl in HF. Furthermore, A-RDN enhanced GLP-1-induced diuresis and natriuresis as well as T-RDN, suggesting that afferent renal nerve is essential for regulating the response of increase in RSNA to GLP-1 described above.

We observed that GLP-1 increased MAP and HR in both HF and Sham rats. These results are consistent with previous reports in which GLP-1 and GLP-1R agonists cause transient increases in MAP and HR in both normotensive and hypertensive rodents [10, 13, 17, 57, 58]. Moreover, we found that increases in MAP and HR by GLP-1 were attenuated by T-RDN or A-RDN in both HF and Sham. The effects of T-RDN and those of A-RDN on MAP and HR were not significantly different between groups. These findings imply that the input from renal afferent to the central nervous system augments sympathetic outflow to the heart and the peripheral vasculatures as well as the kidney in HF and Sham. It should be noted that in current study T-RDN was performed unilaterally while A-RDN was performed bilaterally. Since unilateral T-RDN and bilateral A-RDN had comparable effects on GLP-1 mediated hemodynamic changes, it is possible that unilateral interruption of renal afferent input (limited input) to the central nervous system is sufficient to reduce sympathetic outflow affecting GLP-1 mediated hemodynamic changes. These findings are consistent with a previous study reporting that unilateral T-RDN affects global autonomic balance to restore baroreflex dysfunction associated with HF in rabbits [59]. To further assess the additive effects of bilateral versus unilateral T-RDN on GLP-1 mediated hemodynamic changes remains to be examined.

It has been reported that GLP-1 can improve energy efficiency by increasing glucose assimilation in the heart, which promote cardiac performance [60, 61]. In current study we have demonstrated that GLP-1 increased BP and HR accompanied with diuresis and natriuresis. One possible interpretation could be that GLP-1 promotes cardiac performance to increase BP and HR inducing pressure natriuresis. On the other hand, RDN enhances diuresis despite decreasing BP response to GLP-1, suggesting that GLP-1 mediated diuresis and natriuresis are mainly regulated by renal sympathetic nerve activity rather than pressure natriuresis affected by cardiac performance.

Effect of GLP-1 on HF has been a subject of active debate [6–9]. In animal models of HF, GLP-1R agonists increase myocardial glucose uptake [62], stroke volume and LVEF, accompanied by decreasing LVEDP and systemic vascular resistance [63]. Additionally, diuretic and natriuretic effects of GLP-1 seem to be beneficial for HF. However, these positive benefits in animal studies have apparently not been translated into likely beneficial clinical outcomes. Cardiovascular outcome studies of GLP-1R agonists do not affect the rate of heart failure hospitalization, while decreasing the rate of myocardial infarction and stroke [3–5]. On the other hand, there are some clinical studies suggesting the association between GLP-1 related medicines and the increased risk of HF hospitalization [64–67]. In diabetic patients with advanced HF and reduced LVEF < 40%, GLP-1R agonist had a tendency to increase the risk of rehospitalization for HF [66]. Other studies showed that dipeptidyl peptidase 4 inhibitors, GLP-1 enhancers, significantly increased the risk of hospitalization for HF [64, 65]. From the point of view of clinical relevance, pathophysiological responses to GLP-1 demonstrated in current study including acute increases in RSNA, MAP and HR and decreases in diuresis and natriuresis, all features that are likely to be unfavorable and might contribute to a greater risk for HF.

A previous study has shown that the combination of GLP-1R agonist and angiotensin receptor blocker (ARB) increases natriuresis compared to GLP-1R agonist alone in Otsuka Long–Evans Tokushima Fatty rats, a model of obese type 2 diabetes [68]. Further, a clinical study has demonstrated that the combination of GLP-1R agonist and sodium–glucose cotransporter 2 (SGLT2) inhibitors significantly reduces the risk of all cause-mortality and improves renal function, compared to GLP-1R agonist alone in type 2 diabetic patients [69]. ARBs and SGLT2 inhibitors could exert sympathoinhibitory effects in HF [70–73] and thus restore the diuretic and natriuretic effects of GLP-1. These results are similar to the effects of RDN demonstrated in current study, providing additional insight into safety and benefit of combination treatment with GLP-1 related medicines for diabetic HF patients.

## Conclusions

We have shown that GLP-1R expression in renal pelvis densely innervated by afferent renal nerves was enhanced in HF. The excessive activation of neural circuitry of afferent and efferent renal nerves in HF suppressed diuretic and natriuretic responses to GLP-1. T-RDN or A-RDN restored diuretic and natriuretic effects of GLP-1 (Fig. 8) and has potential therapeutic implication for use of the new catheter based renal denervation technique in diabetic HF patients [74–76].

## Data Availability

All data generated or analyzed during this study are included in this published article.

## Abbreviations

GLP-1: Glucagon-like peptide-1
GLP-1R: GLP-1 receptor
HF: Heart failure
LVEDD: Left ventricular end-diastolic dimension
LVESD: Left ventricular end-systolic dimension
FS: Fractional shortening
EF: Ejection fraction
LVEDV: Left ventricular end-diastolic volume
LVESV: Left ventricular end-systolic volume
SV: Stroke volume
CO: Cardiac output
LVEDP: Left ventricular end-diastolic pressure
LVESP: Left ventricular end-systolic pressure
+dP/dt: Maximal slope of systolic pressure increment
−dP/dt: Maximal slope of diastolic pressure decrement
LV: Left ventricle
ARNA: Afferent renal nerve activity
MAP: Mean arterial pressure
HR: Heart rate
qRT-PCR: Quantitative reverse-transcription polymerase chain reaction
TBST: Tris-buffered saline with tween 20
RSNA: Renal sympathetic nerve activity
T-RDN: Total renal denervation
A-RDN: Afferent renal denervation
CGRP: Calcitonin gene-related peptide
TH: Tyrosine hydroxylase
CrCl: Creatinine clearance
ERNA: Efferent renal nerve activity
PVN: Paraventricular nucleus
ARB: Angiotensin receptor blocker
SGLT2: Sodium–glucose cotransporter 2
